# Unveiling the potential of lactic acid bacteria from Algerian dromedary camel milk: diversity, technological applications, and antimicrobial insights

**DOI:** 10.3389/fnut.2025.1647344

**Published:** 2025-09-18

**Authors:** Bilal Latreche, Esma Bendjama, Lotfi Loucif, Ibtissem Sanah, Mohammed Messaoudi, Chawki Bensouici, Fairouz Djeghim, Khawla Kerbab, Jean-Marc Rolain, Maria D’Elia, Luca Rastrelli, Samira Becila

**Affiliations:** ^1^Laboratoire de recherche en Sciences Alimentaires, Formulation, Innovation, Valorisation et Intelligence Artificielle (SAFIVIA), Institut de la Nutrition, de l’Alimentation et des Technologies Agro-Alimentaires (INATAA), Université Frères Mentouri Constantine 1, Constantine, Algeria; ^2^Laboratoire de Génie Biologique, Valorisation et Innovation des Produits Agro-alimentaires (LGBVIPA), Institut des Sciences et des Techniques Appliquées (ISTA), Ain M’lila, Université Larbi Ben M’hidi, Oum El Bouaghi, Algeria; ^3^Département de Technologie Alimentaire, Institut des Sciences Vétérinaires et des Sciences Agronomiques, Université Batna 1, Batna, Algeria; ^4^Laboratoire de Biotechnologie des Molécules Bioactives et de la Physiopathologie Cellulaire (LBMBPC), Faculté des Sciences de la Nature et de la Vie, Université Batna 2, Batna, Algeria; ^5^Laboratoire de Chimie, Ecole Normale Supérieure – Kouba, Alger, Algeria; ^6^Biotechnology Research Center (CRBt), Constantine, Algeria; ^7^Équipe FNPAA, Laboratoire de Nutrition et Technologie Alimentaire (LNTA), Institut de la Nutrition, de l’Alimentation et des Technologies Agro-Alimentaires (INATAA), Université Frères Mentouri Constantine 1, Constantine, Algeria; ^8^IHU Méditerranée Infection, MEPHI, Faculté de Médecine et de, Pharmacie Aix Marseille Université, Marseille, France; ^9^Department of Pharmacy, University of Salerno, Salerno, Italy; ^10^National Biodiversity Future Center (NBFC), Palermo, Italy; ^11^Dipartimento di Scienze della Terra e del Mare, University of Palermo, Palermo, Italy; ^12^Laboratoire de Recherche en Biotechnologie et Qualité des Aliments (BIOQUAL), Institut de la Nutrition, de l’Alimentation et des Technologies Agro-Alimentaires (INATAA), Université Frères Mentouri Constantine 1, Constantine, Algeria

**Keywords:** lactic acid bacteria (LAB), dromedary camel milk, technological properties, antimicrobial activity, MALDI-TOF MS, microbial diversity

## Abstract

**Background:**

Lactic acid bacteria (LAB) play a central role in the food industry due to their ability to produce beneficial metabolites and enhance the technological and sensory qualities of fermented products. Additionally, they contribute to human health by supporting immune function and maintaining gut microbiota balance through probiotic effects. This study aimed to isolate and characterize LAB from dromedary camel milk (DCM) collected in semi-arid regions of Algeria, evaluating their technological functionalities and antimicrobial activities.

**Methods:**

A total of 31 LAB strains were isolated from raw DCM samples. Strains were identified using MALDI-TOF MS and characterized for acidification kinetics, lipolytic, proteolytic, and amylolytic activities, exopolysaccharide (EPS) and acetoin production, and antimicrobial properties against common foodborne pathogens.

**Results:**

Four species were identified, with *Enterococcus italicus* reported for the first time in this environment. Significant inter-strain variability (*p* < 0.0001) was observed in all tested properties. Three strains (*BLC9*, *BLC12*, *BLC14*) acidified milk rapidly to pH 4.6 within 12 h. Proteolytic activity was detected in 87.10% of strains, while EPS and acetoin were produced by 29.03 and 48.39%, respectively. Lipolytic and amylolytic activities were generally weak. Notably, 74.19% of the strains exhibited antimicrobial activity, inhibiting at least one pathogen, with inhibition zones varying significantly (*p* < 0.0001).

**Conclusion:**

Dromedary camel milk from Algerian semi-arid regions represents a rich source of LAB strains with promising technological and antimicrobial potential. These native isolates could be further developed for use in additive-free fermented foods and natural biopreservation systems, supporting sustainable and functional food innovation.

## 1 Introduction

The production of a wide range of fermented foods relies on the use of starter cultures—microbial consortia introduced into raw substrates to initiate and direct fermentation. Among these, lactic acid bacteria (LAB) play a pivotal role. Historically, being used for millennia in food preservation, LAB enhance shelf life and microbiological safety primarily through acidification and competitive exclusion of spoilage organisms and pathogens (1, 2).

Beyond their preservative functions, LAB are now widely recognized for their functional and technological potential. Through their diverse metabolic activities, LAB synthesize an array of bioactive metabolites that influence the texture, flavor, and nutritional quality of fermented foods, while also contributing to host health (3, 4). Their status as Generally Recognized As Safe (GRAS) and Qualified Presumption of Safety (QPS) further supports their broad acceptance and utilization across the food industry (5).

LAB possess a rich enzymatic repertoire, including proteases, peptidases, ureases, lipases, amylases, esterases, and phenol oxidases, enabling the hydrolysis of complex substrates such as polysaccharides, proteins, and lipids. They are also capable of metabolizing dietary fibers and aromatic precursors, producing diverse secondary metabolites such as short-chain fatty acids, biogenic amines, bacteriocins, vitamins, exopolysaccharides (EPS), organic acids, and carbon dioxide. These attributes make LAB highly relevant to both food quality improvement and biological safety enhancement (6–8).

The exploration of LAB from non-conventional, underexplored habitats represents a promising frontier in microbial biotechnology, with potential applications in the development of novel functional foods and natural biopreservatives (9).

The dromedary camel (*Camelus dromedarius*), well-adapted to arid and semi-arid climates, produces milk notable for its rich composition in proteins, vitamins, minerals, and bioactive molecules with reported health benefits (10, 11). Camel milk has also gained attention as a potential source of unique microbial strains with functional and technological relevance (12).

In Algeria, dromedary camel milk (DCM) is traditionally consumed raw or fermented by nomadic populations. Its unique biochemical profile, including high levels of lysozyme, lactoperoxidase, lactoferrin, and LAB-produced bacteriocins, suggests a high antimicrobial potential (13). Despite this, the LAB microbiota of DCM remains relatively underexplored.

In this study, 31 strains of LAB were isolated from raw dromedary milk from semi-arid regions of Algeria and identified by both phenotypic methods and MALDI-TOF MS mass spectrometry. These strains were then evaluated for their technological and antimicrobial potential against seven pathogens. The objective was to identify promising candidate strains for future applications in food biotechnology and biopreservation. This atypical dairy matrix, rich in bioactive compounds, provides a favorable ecological niche for selecting strains of interest.

## 2 Materials and methods

### 2.1 Origin and collection of milk samples

**Figure 1:**
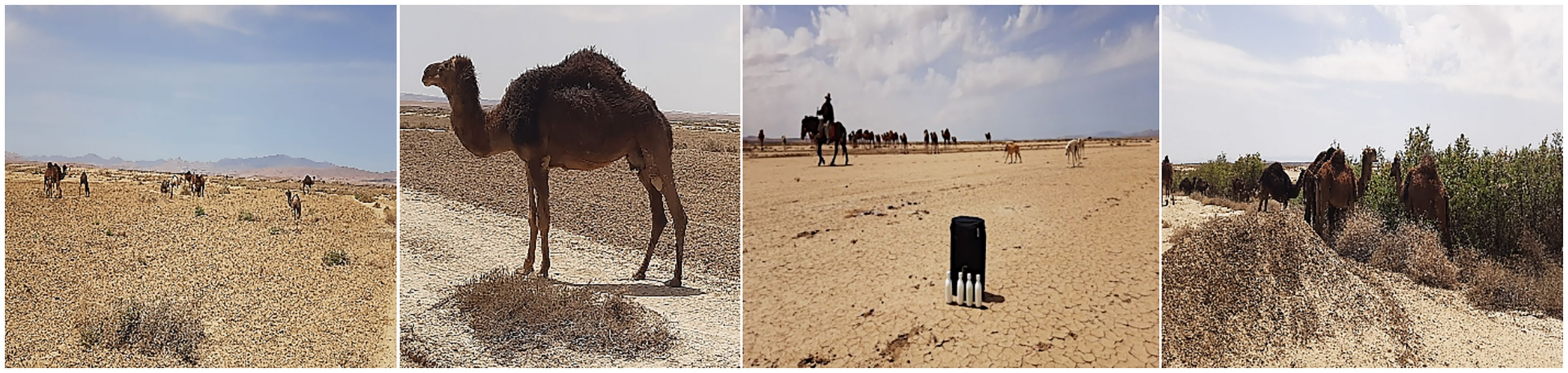
Collection of dromedary camel milk (DCM) samples from semi-arid regions of Algeria.

To begin the sample collection process, 10 (*n* = 10) milk samples were taken from dromedary camels (*Camelus dromedarius*). These animals were sourced from farms located in a semi-arid environment, specifically Khattouti Sed El Djir, Aïn El Hadjel, Maarif, Chellal, and Ouled Madhi, within the M’Sila province of central Algeria. The camels were selected based on their health status and lactation period on each farm (Figure 1; Table 1). This region lies at the confluence of the Tell Atlas and Hodna Basin and represents a typical semi-arid ecosystem. Sampling was conducted over a three-year period, from May 2019 to May 2022. To ensure sample representativeness, pooled milk samples were obtained from multiple camels within each herd. Milking was carried out manually using traditional practices. For each sampling event, approximately 1 liter of raw milk was aseptically collected into sterile glass bottles. Samples were immediately stored in insulated containers with ice packs and transported to the laboratory under chilled conditions for further microbiological and physicochemical analyses (14).

**Table 1:** Characteristics of dromedary camel milk (DCM) samples collected in the semi-arid regions of Algeria.

Samples	Regions	Number of camels	Approximate age	Date	Season
S.01	Khattouti Sed El Djir	8	4 to 6 years	19/05/2019	Summer
S.02	Maarif	6	6 to 10 years	15/07/2019	Summer
S.03	Maarif	6	6 to 10 years	28/07/2019	Summer
S.04	Aïn El Hadjel	10	5 to 9 years	06/08/2019	Summer
S.05	Chellal	4	4 to 12 years	26/03/2020	Spring
S.06	Chellal	6	4 to 12 years	04/04/2020	Spring
S.07	Khattouti Sed El Djir	7	5 to 8 years	15/01/2021	Winter
S.08	Ouled Madhi	8	4 to 6 years	26/02/2021	Winter
S.09	Aïn El Hadjel	12	7 to 12 years	14/03/2022	Spring
S.10	Ouled Madhi	5	5 to 8 years	22/05/2022	Summer

### 2.2 Isolation, purification, and preservation of LAB isolates

LAB were isolated from dromedary camel milk (DCM) by spread-plating serial dilutions (10^−2^ to 10^−7^) onto M17 agar (Conda, Madrid, Spain) and de Man, Rogosa and Sharpe (MRS) agar (Merck Millipore, Germany). Plates were incubated at 30 °C for 48–72 h under aerobic conditions. Colonies exhibiting typical LAB morphology were selected, sub-cultured for purification, and preserved for long-term storage. Pure isolates were suspended in a cryoprotective mixture composed of culture broth and glycerol, and stored at −80 °C in sterile Eppendorf tubes to maintain viability (15).

### 2.3 Phenotypic, physiological, and biochemical characterization

LAB isolates were initially screened based on phenotypic traits, including microscopic morphology and catalase activity, following standard protocols (16, 17). Only isolates that were Gram-positive, catalase-negative, and non-motile were retained as presumptive LAB candidates. Genus-level differentiation was subsequently conducted by evaluating glucose fermentation patterns, growth in 6.5% NaCl, tolerance to alkaline pH (9.6), and the ability to grow at different temperatures (10 °C, 15 °C, and 45 °C). Additional biochemical characterization was performed using the arginine dihydrolase test (18–20).

### 2.4 Bacterial species identification by MALDI-TOF MS

The identification of the LAB strains isolated from camel milk was performed by MALDI-TOF mass spectrometry, following the protocol described by Seng et al. (21).

#### 2.4.1 Matrix preparation

A saturated solution of *α*-cyano-4-hydroxycinnamic acid (HCCA) was prepared by mixing 250 μL of 10% trifluoroacetic acid (TFA), 250 μL of HPLC-grade water, and 500 μL of HPLC-grade acetonitrile in a 1.5 mL microcentrifuge tube under a chemical fume hood. The mixture was vortexed vigorously, sonicated for 10 min, and centrifuged at 13,000 × *g* for 5 min. The resulting supernatant was transferred to a fresh 1.5 mL tube and used as the working matrix solution.

#### 2.4.2 Sample deposition on the MALDI-TOF MS target

Fresh bacterial colonies were picked using sterile pipette tips and applied in a thin, homogeneous layer onto designated spots of a stainless-steel MALDI target plate (Bruker Daltonics). Each isolate was spotted in triplicate. In parallel, matrix-only spots (negative controls) and reference strains (positive controls) were included. Subsequently, 1.5 μL of the prepared matrix solution was added to each spot and allowed to dry at room temperature to ensure complete co-crystallization of matrix and sample.

#### 2.4.3 Spectral acquisition and species identification

Once dried, the MALDI target was introduced into the Microflex LTII MALDI-TOF MS spectrometer (Bruker Daltonics, Bremen, Germany). Spectra were acquired using the manufacturer’s standard settings. Identification was performed using the Bruker Biotyper software by matching the protein mass spectra to the reference database.

Identification scores were interpreted according to Bruker’s standard criteria:

- Score ≥ 2.0: Secure identification at the species level
- Score 1.7–1.99: Probable identification at the genus level
- Score < 1.7: Unreliable identification

Only strains with score values ≥ 2.0 were considered correctly identified at the species level.

### 2.5 Evaluation of the technological properties of LAB strains

#### 2.5.1 Acidification activity

LAB isolates were cultivated in MRS or M17 broth (according to their original isolation medium) and incubated at 30°C for 18–24 h. Prior to testing, cultures were standardized to an optical density (OD) of approximately 1.00 at 600 nm using a spectrophotometer (Helios Epsilon, Thermo Fisher Scientific, United States).

Each standardized culture (1% v/v) was inoculated into 200 mL of ultra-high temperature (UHT) skimmed milk. The inoculated samples were incubated at 30°C, and acidification kinetics were assessed by monitoring pH at 0, 2, 4, 6, 8, 24, and 48 h using a calibrated pH meter (SevenCompact S220, Mettler Toledo, Switzerland), as described by Domingos-Lopes et al. (22).

The acidification rate was calculated as:


$$\mathrm{\Delta pH}={\mathrm{pH}}_{\text{initial}}-{\mathrm{pH}}_{\text{measured}}$$

Where:

- pH_initial_: Initial pH of the UHT milk
- pH_measured_: pH recorded after the incubation period

#### 2.5.2 Lipolytic activity

Lipolytic activity was assessed using triglyceride agar supplemented with 1% Tween 20. Sterile Whatman paper discs were placed on the surface of the solidified medium and each disc was inoculated with 10 μL of a log-phase LAB culture. Plates were incubated at 30°C for 24–48 h. Lipolytic activity was evidenced by the formation of a clear halo surrounding the disc, indicating enzymatic hydrolysis of triglycerides (23, 24).

#### 2.5.3 Proteolytic activity

Proteolytic activity was evaluated qualitatively using an agar diffusion assay. Sterile Whatman paper discs were placed on the surface of plate count agar (PCA; Scharlau, Barcelona, Spain) supplemented with 1% (w/v) UHT skimmed milk powder. Each disc was inoculated with 10 μL of a log-phase LAB culture. Plates were incubated at 30 °C for 3 to 5 days. Proteolytic activity was indicated by the appearance of clear zones around the discs, corresponding to casein hydrolysis. The diameter of the lysis zones was measured in millimeters to assess the extent of proteolytic activity (22, 25).

#### 2.5.4 Exopolysaccharide production

Exopolysaccharide (EPS) production was initially screened by streaking log-phase LAB cultures on MSE agar supplemented with 10% (w/v) sucrose. After incubation at 30°C for 48 to 72 h, colonies were examined visually. The presence of large, viscous, and slimy colonies indicated potential EPS producers. To confirm EPS synthesis, strains were further cultured in MRS or M17 broth supplemented with sucrose and incubated at 30 °C for 24 h. The cultures were then centrifuged at 5,000 rpm for 10 min at 4 °C. One milliliter of the supernatant was transferred into a clean tube, and an equal volume of 95% ethanol was added. The formation of an opaque ring at the interface confirmed EPS production (26).

#### 2.5.5 Amylase production potential

The amylolytic activity of LAB isolates was assessed using a starch hydrolysis assay based on the disk diffusion method. Sterile Whatman paper discs were inoculated with 10 μL of log-phase cultures and placed onto starch agar plates. After 24 h of incubation at 30°C, plates were flooded with Lugol’s iodine solution and allowed to react for 15–30 min to form a starch–iodine complex. The presence of a clear halo around the inoculated discs indicated starch hydrolysis and thus positive amylolytic activity (27, 28).

#### 2.5.6 Acetoin production capacity

Acetoin production, indicative of flavoring potential, was evaluated using the Voges–Proskauer (VP) test. LAB strains were cultured in Clark and Lubs medium and incubated at 30°C for 24 h. Following incubation, 2 mL of the culture was transferred to a sterile tube, and 0.5 mL of 16% sodium hydroxide (VP1) and 0.5 mL of 6% *α*-naphthol (VP2) in absolute ethanol were added sequentially. The tubes were gently agitated and left at room temperature for 5–10 min. A positive acetoin reaction was indicated by the formation of a pink ring at the surface of the medium (23, 29).

### 2.6 Antimicrobial activity assay

The antimicrobial activity of LAB isolates was determined using the Kirby–Bauer disk diffusion method. Target pathogenic strains included *Staphylococcus aureus* (ATCC 25923), *Escherichia coli* (ATCC 25922), *Salmonella enteritidis* (ATCC 13076), *Bacillus cereus* (ATCC 10876), *Listeria monocytogenes* (ATCC 13932), *Pseudomonas aeruginosa* (ATCC 27853), and *Enterobacter cloacae* (ATCC 13047). Mueller–Hinton agar plates were inoculated with each pathogen at 0.5 McFarland standard turbidity. Sterile filter paper discs (6 mm diameter) were impregnated with 10 μL of LAB culture and placed on the agar surface. After drying at room temperature, plates were pre-incubated at 4°C for 4 h to enhance metabolite diffusion, followed by incubation at 30 °C for 24 h. Antimicrobial activity was evaluated by measuring the diameter (mm) of the inhibition zones surrounding each disc (30).

### 2.7 Statistical analysis

Descriptive statistics were initially applied to summarize the data and characterize the phenotypic and functional traits of the LAB isolates. To assess significant differences between mean values of technological and antimicrobial parameters, one-way analysis of variance (ANOVA) was conducted, followed by Tukey’s *post hoc* test for multiple comparisons. Additionally, hierarchical cluster analysis (HCA) using Ward’s method was employed to classify the isolates based on their technological and antibacterial profiles, identifying groups with shared characteristics. All statistical analyses were performed using JMP Trial 17 software (SAS Institute Inc., Cary, NC, United States). Results are presented as mean ± standard deviation (SD), and statistical significance was set at *p* < 0.05.

## 3 Results

### 3.1 Isolation and preliminary characterization

**Figure 2:**
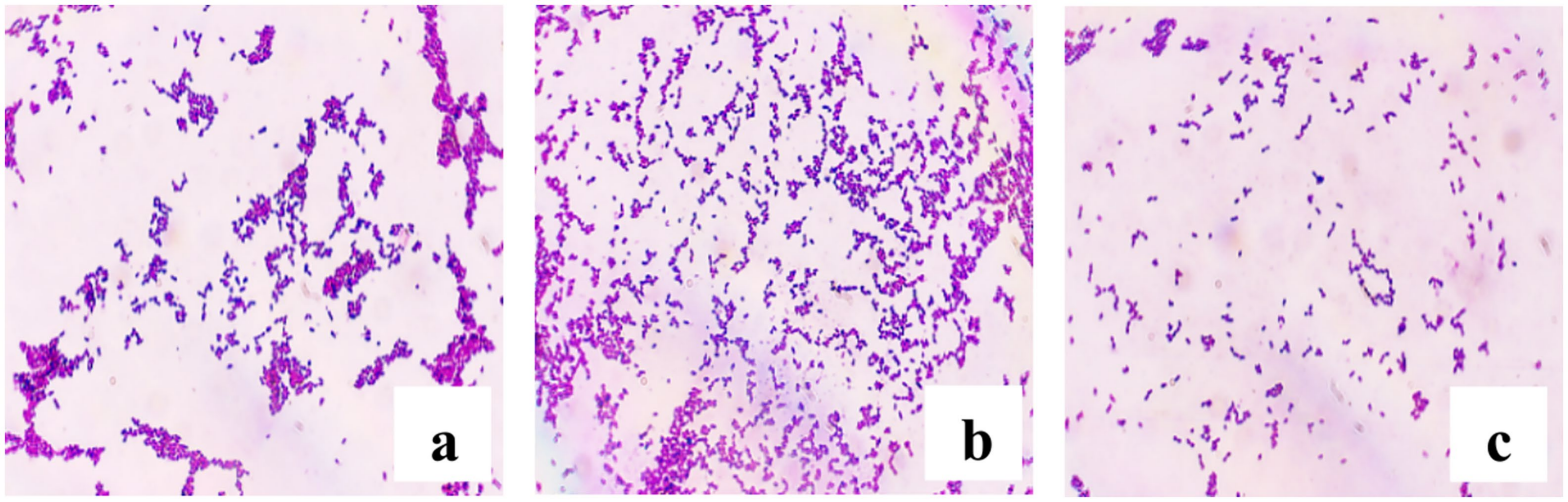
Microscopic observation of Gram-stained LAB isolates (100 × magnification). **(a)** BLC7, **(b)** BLC13, **(c)** BLC16.

A total of 79 presumptive LAB isolates were recovered from DCM samples. Based on preliminary screening (Figure 2), 31 isolates exhibiting typical coccoid morphology were selected for further characterization as potential LAB candidates.

### 3.2 Phenotypic characterization

The selected LAB isolates were assigned to four genera based on phenotypic and biochemical criteria, including cell morphology, glucose fermentation, temperature-dependent growth, pH and salt tolerance, and arginine dihydrolase activity (Table 2).

**Table 2:** Phenotypic characteristics of LAB strains isolated from DCM.

Strain	Morphology	Gram	Catalase	Motility	Tetrads	Growth at 10 °C	Growth at 15 °C	Growth at 45 °C	CO_₂_ production	6.5% NaCl	pH 9.6	Arginine (ADH)
BLC1	Coccus	+	−	−	−	+	ND	−	−	ND	−	ND
BLC2	Coccus	+	−	−	−	ND	ND	+	−	+	+	ND
BLC3	Coccus	+	−	−	−	+	ND	−	−	ND	−	ND
BLC4	Coccus	+	−	−	−	+	ND	−	−	ND	−	ND
BLC5	Coccus	+	−	−	−	+	ND	−	−	ND	−	ND
BLC6	Coccus	+	−	−	−	+	ND	−	−	ND	−	ND
BLC7	Coccus	+	−	−	−	+	ND	−	−	ND	−	ND
BLC8	Coccus	+	−	−	−	+	ND	−	−	ND	−	ND
BLC9	Coccus	+	−	−	−	+	ND	−	−	ND	−	ND
BLC10	Coccus	+	−	−	−	+	ND	−	−	ND	−	ND
BLC11	Coccus	+	−	−	−	+	ND	−	−	ND	−	ND
BLC12	Coccus	+	−	−	−	+	ND	−	−	ND	−	ND
BLC13	Coccus	+	−	−	−	+	ND	−	−	ND	−	ND
BLC14	Coccus	+	−	−	−	+	ND	−	−	ND	−	ND
BLC15	Coccus	+	−	−	ND	ND	ND	ND	+	ND	ND	−
BLC16	Coccus	+	−	−	−	+	ND	−	−	ND	−	ND
BLC17	Coccus	+	−	−	−	+	ND	−	−	ND	−	ND
BLC18	Coccus	+	−	−	−	+	ND	−	−	ND	−	ND
BLC19	Coccus	+	−	−	−	+	ND	−	−	ND	−	ND
BLC20	Coccus	+	−	−	−	+	ND	−	−	ND	−	ND
BLC21	Coccus	+	−	−	−	+	ND	−	−	ND	−	ND
BLC22	Coccus	+	−	−	−	+	ND	−	−	ND	−	ND
BLC23	Coccus	+	−	−	−	+	ND	−	−	ND	−	ND
BLC24	Coccus	+	−	−	−	+	ND	−	−	ND	−	ND
BLC25	Coccus	+	−	−	−	+	ND	−	−	ND	−	ND
BLC26	Coccus	+	−	−	−	ND	ND	+	−	+	+	ND
BLC27	Coccus	+	−	−	−	+	ND	−	−	ND	−	ND
BLC28	Rod	+	−	−	ND	ND	+	ND	−	ND	ND	ND
BLC29	Coccus	+	−	−	−	+	ND	−	−	ND	−	ND
BLC30	Coccus	+	−	−	−	+	ND	−	−	ND	−	ND
BLC31	Coccus	+	−	−	−	+	ND	−	−	ND	−	ND

+, positive result; −, negative result; ND, not determined.

**Figure 3:**
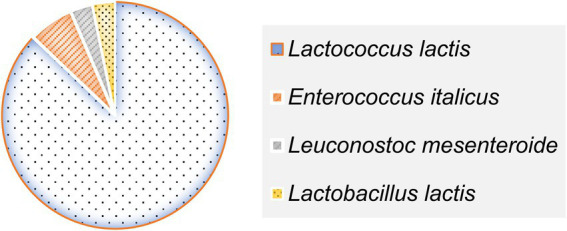
Species distribution of 31 LAB strains isolated from raw DCM samples.

Of the 31 isolates:

- 27 strains were identified as belonging to the *Lactococcus* genus. These strains were tetrad-negative, capable of growth at 10 °C but not at 45 °C, did not produce CO_2_, and were intolerant to alkaline conditions (pH 9.6).
- 2 strains were assigned to the *Enterococcus* genus, characterized by growth at 45 °C, tolerance to 6.5% NaCl and pH 9.6, and absence of tetrads.
- The single *Leuconostoc* isolate produced CO_2_ but tested negative for arginine dihydrolase activity.
- One isolate, identified as *Lactobacillus*, demonstrated growth at 15 °C without CO_2_ production (Figure 3).

These findings reflect a diverse representation of LAB genera with distinctive phenotypic traits adapted to the semi-arid camel milk microbiota.

### 3.3 MALDI-TOF MS identification of LAB isolates

All 31 LAB strains isolated from DCM were successfully identified to the species level using MALDI-TOF MS, based on their peptide mass fingerprint profiles. Identification was performed through spectral comparison against the Bruker reference database, with all log score values exceeding the established reliability threshold of 2.0, thus indicating high-confidence species-level matches (21). The identification scores ranged from 2.12 (*Enterococcus italicus*, strain *BLC26*) to 2.46 (*Lactococcus lactis*, strain *BLC5*) (Table 3). The most prevalent species was *Lactococcus lactis*, which accounted for 87.1% of the isolates (27/31), in strong agreement with prior phenotypic characterization. In addition to this dominant species, two strains (*BLC2* and *BLC26*) were assigned to *Enterococcus italicus,* a species rarely reported in dromedary-derived matrices. One isolate was identified as *Leuconostoc mesenteroides* (*BLC15*), and another as *Lactobacillus lactis* (*BLC28*), underscoring the microbial diversity present in DCM from semi-arid Algerian regions. These results validate the use of MALDI-TOF MS as a rapid and accurate tool for LAB identification in complex matrices such as camel milk, as previously demonstrated in dairy microbial ecology studies (31, 32).

**Table 3:** Species identification results obtained by MALDI-TOF MS for the 31 LAB strains isolated from DCM collected in the semi-arid regions of Algeria.

Isolate	Identification result	Score
BLC1	*Lactococcus lactis*	2.37
BLC2	*Enterococcus italicus*	2.32
BLC3	*Lactococcus lactis*	2.44
BLC4	*Lactococcus lactis*	2.38
BLC5	*Lactococcus lactis*	2.46
BLC6	*Lactococcus lactis*	2.32
BLC7	*Lactococcus lactis*	2.35
BLC8	*Lactococcus lactis*	2.44
BLC9	*Lactococcus lactis*	2.32
BLC10	*Lactococcus lactis*	2.37
BLC11	*Lactococcus lactis*	2.39
BLC12	*Lactococcus lactis*	2.27
BLC13	*Lactococcus lactis*	2.29
BLC14	*Lactococcus lactis*	2.32
BLC15	*Leuconostoc mesenteroides*	2.24
BLC16	*Lactococcus lactis*	2.30
BLC17	*Lactococcus lactis*	2.33
BLC18	*Lactococcus lactis*	2.42
BLC19	*Lactococcus lactis*	2.38
BLC20	*Lactococcus lactis*	2.37
BLC21	*Lactococcus lactis*	2.29
BLC22	*Lactococcus lactis*	2.29
BLC23	*Lactococcus lactis*	2.23
BLC24	*Lactococcus lactis*	2.41
BLC25	*Lactococcus lactis*	2.34
BLC26	*Enterococcus italicus*	2.12
BLC27	*Lactococcus lactis*	2.23
BLC28	*Lactobacillus lactis*	2.32
BLC29	*Lactococcus lactis*	2.27
BLC30	*Lactococcus lactis*	2.37
BLC31	*Lactococcus lactis*	2.29

A log score value ≥2.0 indicates reliable identification at the species level.

### 3.4 Acidification activity

**Figure 4:**
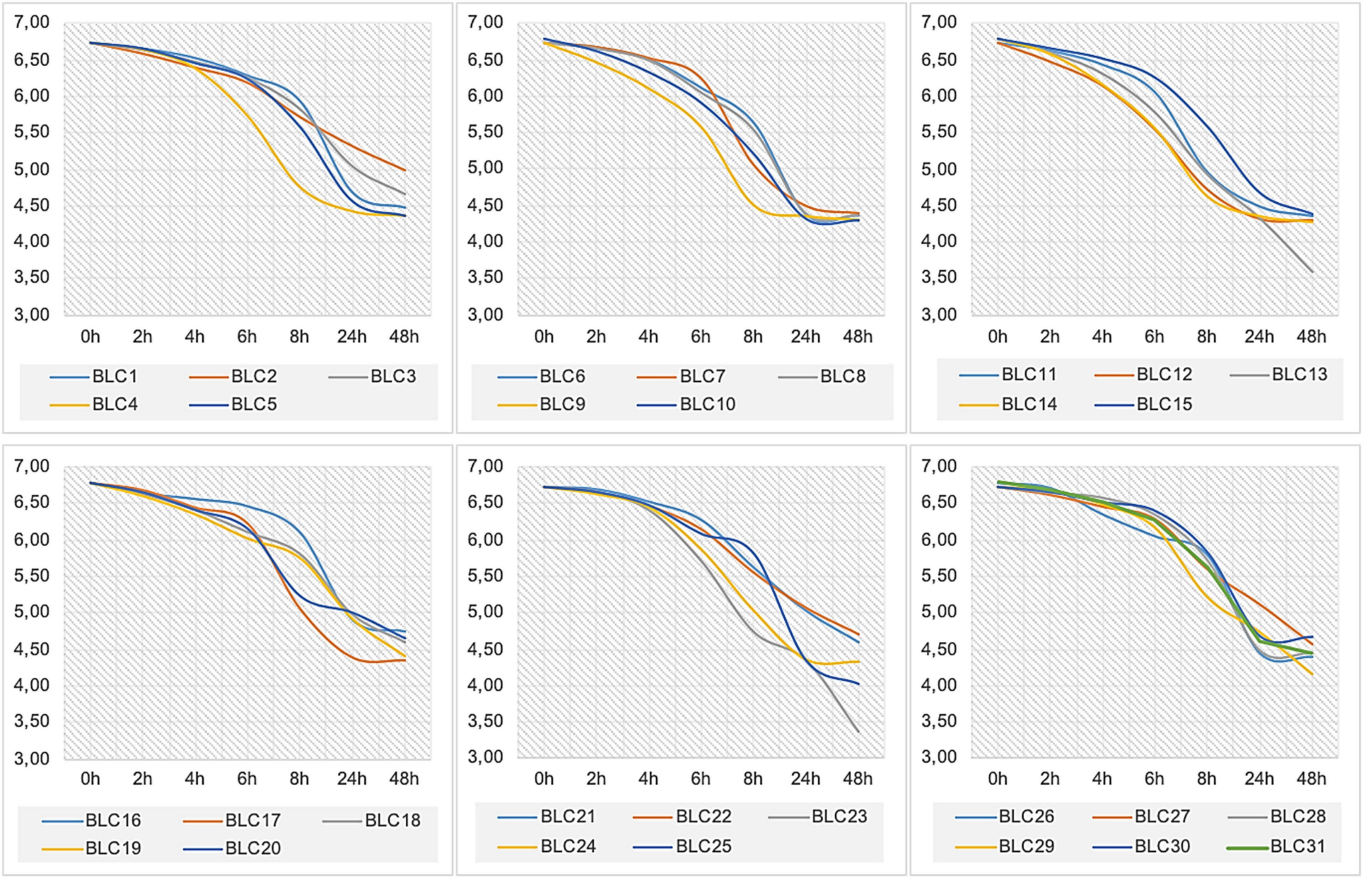
Acidification activity of LAB strains in UHT skimmed milk at 30°C.

The acidification profiles of the 31 LAB strains revealed marked heterogeneity in acidification kinetics (Figure 4; *p* < 0.0001). All isolates were able to lower milk pH during the first 6 h of fermentation at 30°C, although none reached a pH below 5.0 within this initial phase.

After 24 h, acidification intensified across all strains, with ΔpH values ranging from 1.42 (*BLC2*) to 2.47 (*BLC10* and *BLC13*). The majority of strains reduced the milk pH to values between 4.0 and 5.0, indicating effective acid production. At 48 h, ΔpH ranged from 1.57 (*BLC29*) to 3.20 (*BLC13*), confirming sustained acidification potential. Notably, strains *BLC9*, *BLC12*, and *BLC14* exhibited rapid acidification, reducing the pH to 4.6 in less than 12 h. Most other isolates were classified as moderate acidifiers, reaching this threshold between 12 and 48 h. Conversely, strains *BLC2*, *BLC16*, *BLC22*, and *BLC29* demonstrated slower kinetics, requiring more than 48 h to reach pH 4.6, consistent with previously described acidification profiles for less active LAB strains (33).

### 3.5 Lipolytic activity

**Figure 5:**
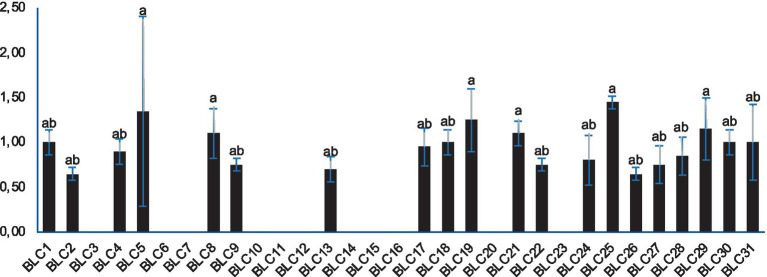
Lipolytic activity of LAB strains on Tween-20 agar. Bacterial strains designated with the same letter do not exhibit statistically significant differences (*p* > 0.05).

Lipolytic activity was detected in 64.5% (20 out of 31) of the LAB isolates when cultured on Tween-20-supplemented agar. The presence of clear halos around inoculated disks confirmed enzymatic hydrolysis of triglycerides, with statistically significant differences observed in the diameters of lysis zones (*p* < 0.0001; Figure 5). The most pronounced lipolytic activity was recorded for strain *BLC25*, which produced the largest halo (1.45 ± 0.07 cm). In contrast, 34.5% of the isolates, mainly identified as *Lactococcus lactis*, showed no detectable lipolytic activity under the tested conditions.

### 3.6 Proteolytic activity

**Figure 6:**
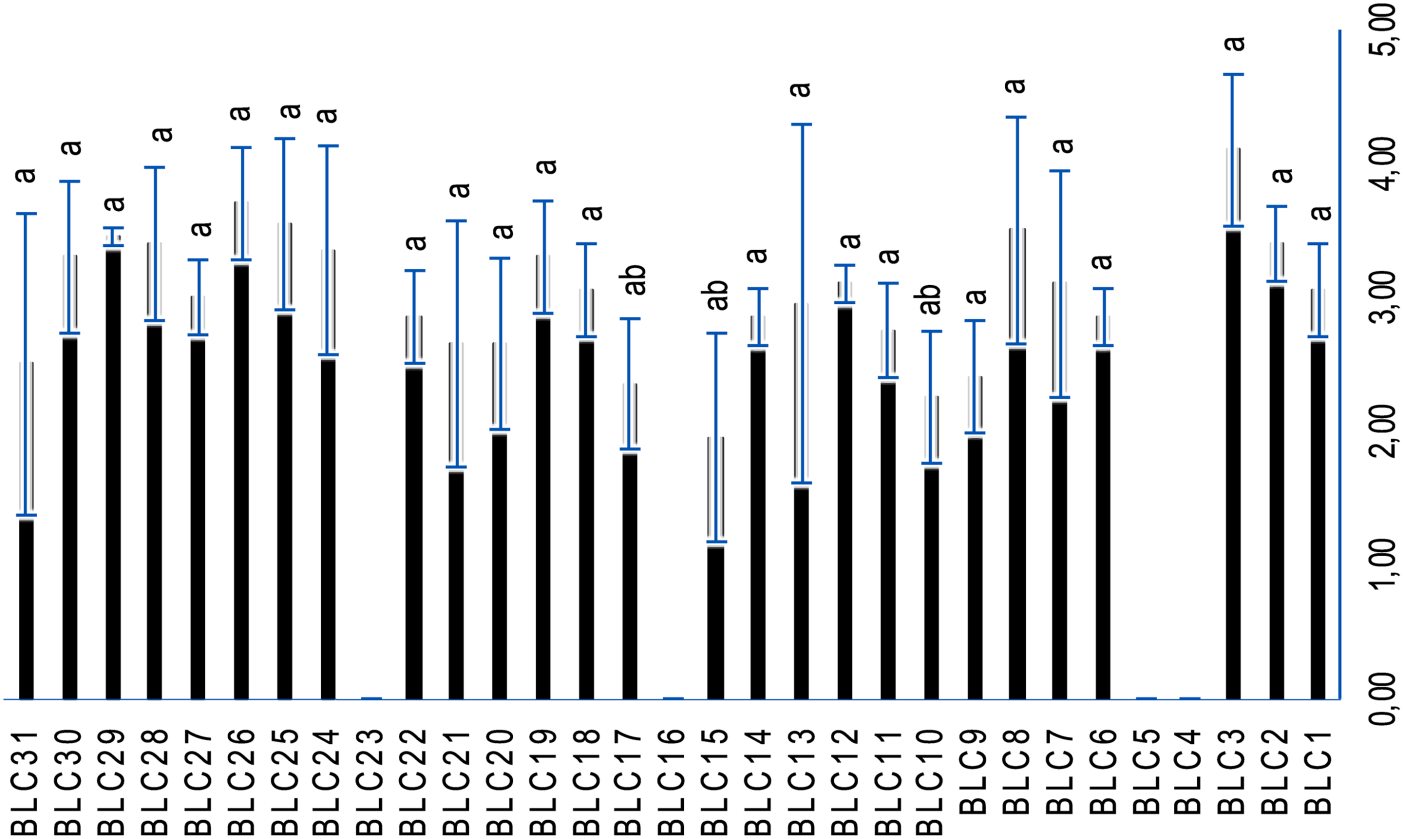
Proteolytic activity of LAB strains on skimmed milk-enriched PCA. Bacterial strains designated with the same letter do not exhibit statistically significant differences (*p* > 0.05).

Proteolytic activity varied significantly among the LAB isolates, with a high degree of inter-strain variability (*p* < 0.0001; Figure 6). A total of 87.1% of the strains exhibited measurable proteolytic activity, as indicated by the formation of clear hydrolysis zones on skimmed milk-enriched agar. Only four strains, *BLC4*, *BLC5*, *BLC16*, and *BLC23*, did not exhibit any proteolytic activity under the conditions tested, highlighting notable functional diversity within the LAB collection.

### 3.7 Exopolysaccharide production

Exopolysaccharide (EPS) production was observed in 29.0% of the LAB isolates when cultured on MSE agar supplemented with sucrose (Table 4). Among these, 22.6% demonstrated low EPS production, while only one strain (*BLC15*) exhibited moderate production levels. The majority of isolates (70.9%) did not produce detectable amounts of EPS under the tested conditions. These findings highlight limited but variable EPS biosynthesis potential within the LAB collection, which may influence the textural and rheological properties of fermented dairy matrices.

**Table 4:** Technological activities of LAB strains isolated from DCM in semi-arid regions of Algeria.

Strain	EPS production^1^	Amylase production^2^ (cm)	Acetoin production^3^
BLC1	−	−	+
BLC2	−	−	+
BLC3	+	−	−
BLC4	−	−	−
BLC5	−	−	−
BLC6	−	−	+
BLC7	−	−	−
BLC8	+	−	−
BLC9	−	−	+
BLC10	−	−	+
BLC11	−	−	−
BLC12	+	−	−
BLC13	−	−	−
BLC14	−	−	−
BLC15	++	−	−
BLC16	−	−	−
BLC17	−	−	−
BLC18	−	−	−
BLC19	−	−	+
BLC20	−	−	+
BLC21	+	−	−
BLC22	+	−	+
BLC23	−	1.20 ± 0.14ᵃᵇ	+
BLC24	−	−	+
BLC25	−	−	+
BLC26	−	−	+
BLC27	−	−	−
BLC28	−	−	+
BLC29	+	0.85 ± 0.07ᵇ	+
BLC30	+	1.35 ± 0.64ᵃ	+
BLC31	+++	−	−

^1^EPS production: (+) Low, (++) Moderate, (+++) High, (−) No activity. ^2^Amylase production: clear zone diameter (cm); (−) Negative test; (Mean ± SD). ^3^Acetoin production: (+) Positive test, (−) Negative test. Values with different superscript letters differ significantly (*p* < 0.05).

### 3.8 Amylolytic activity

Amylolytic activity was detected in only 9.7% of the isolates (*BLC23*, *BLC29*, and *BLC30*), all identified as *Lactococcus lactis* (Table 4). These strains exhibited weak activity, with halo diameters of 1.20 ± 0.14 cm, 0.85 ± 0.07 cm, and 1.35 ± 0.64 cm, respectively. The remaining 90.3% of the LAB isolates showed no detectable starch-degrading activity under the tested conditions, suggesting limited amylolytic potential within this collection.

### 3.9 Acetoin production

Acetoin production was detected in 48.4% of the LAB isolates based on the Voges-Proskauer test (Table 4). The remaining 51.6% of strains tested negative for this metabolic trait. A highly significant inter-strain variability was observed in acetoin production capacity (*p* < 0.0001), indicating functional diversity within the collection. This property is of particular interest for its contribution to flavor development in fermented dairy products.

### 3.10 Antimicrobial activity

The antimicrobial potential of the LAB isolates displayed substantial heterogeneity, with statistically significant differences in inhibitory activity among strains (*p* < 0.0001). Over 67% of the isolates effectively inhibited *Staphylococcus aureus*, producing inhibition zones ranging from 8.0 ± 1.4 mm to 22.0 ± 2.8 mm (Table 5).

**Table 5:** Antimicrobial activities of LAB strains isolated from DCM in semi-arid regions of Algeria (Mean ± SD).

Antibacterial activity^1^							
Indicator strains							
Strain	*Staphylococcus aureus*	*Escherichia coli*	*Salmonella enteritidis*	*Bacillus cereus*	*Listeria monocytogenes*	*Pseudomonas aeruginosa*	*Enterobacter cloacae*
BLC1	13.0 ± 1.4^bcd^	-	-	-	-	8.0 ± 1.4^bc^	7.0 ± 0^b^
BLC2	12.0 ± 1.4^cd^	-	8.5 ± 0.7^ab^	-	11 ± 5.7^a^	8.0 ± 0^bc^	8.5 ± 0.7^ab^
BLC3	11.0 ± 1.4^cd^	-	-	-	-	7.5 ± 0.7^bc^	9.5 ± 0.7^a^
BLC4	-	-	-	-	-	-	-
BLC5	9.5 ± 0.7^cd^	-	-	-	-	7.0 ± 0^c^	-
BLC6	15.0 ± 1.4^bc^	-	7.0 ± 0^c^	-	-	8.0 ± 1.4^bc^	7.5 ± 0.7^ab^
BLC7	12.0 ± 1.4^cd^	-	-	-	-	-	7.5 ± 0.7^ab^
BLC8	12.5 ± 0.7^bcd^	-	-	-	-	8.5 ± 0.7^abc^	8.0 ± 0^ab^
BLC9	-	-	-	-	-	-	9.5 ± 0.7^a^
BLC10	8.0 ± 1.4^d^	-	-	-	-	-	9.0 ± 0^ab^
BLC11	12.0 ± 2.8^cd^	-	-	-	-	9.5 ± 0.7^ab^	-
BLC12	8.5 ± 0.7^d^	-	-	-	-	9.0 ± 0^abc^	9.5 ± 0.7^a^
BLC13	12.0 ± 1.4^cd^	7.5 ± 0.7^b^	-	-	-	-	-
BLC14	11.0 ± 1.4^cd^	8.0 ± 1.4^b^	8.0 ± 0^bc^	-	-	-	8.0 ± 1.4^ab^
BLC15	-	-	-	-	-	-	-
BLC16	-	8.5 ± 0.7^b^	8.5 ± 0.7^ab^	-	-	8.0 ± 1.4^bc^	-
BLC17	-	-	-	-	-	-	-
BLC18	13.5 ± 0.7^bcd^	7.5 ± 0.7^b^	-	-	9.0 ± 1.4^a^	-	-
BLC19	15.0 ± 1.4^bc^	-	7.0 ± 0^c^	-	9.0 ± 1.4^a^	-	-
BLC20	12.5 ± 0.7^bcd^	8.0 ± 0^b^	9.0 ± 1.4^ab^	-	9.5 ± 0.7^a^	10.5 ± 0.7^a^	7.0 ± 0^b^
BLC21	-	-	-	-	-	-	-
BLC22	13.5 ± 2.1^bcd^	-	-	-	8.5 ± 0.7^a^	8.0 ± 0^bc^	8.5 ± 0.7^ab^
BLC23	-	-	-	-	-	-	-
BLC24	15.5 ± 4.9^bc^	9.0 ± 0^b^	-	8.5 ± 0.7^a^	9.0 ± 1.4^a^	9.5 ± 0.7^ab^	8.5 ± 0.7^ab^
BLC25	12.5 ± 2.1^bcd^	9.0 ± 1.4^b^	-	9.0 ± 1.4^a^	8.5 ± 2.1^a^	7.5 ± 0.7^bc^	8.0 ± 1.4^ab^
BLC26	-	-	-	-		-	-
BLC27	22.0 ± 2.8^a^	-	8.5 ± 0.7^ab^	-	-	-	-
BLC28	13.5 ± 0.7^e^	14.5 ± 2.1^a^	-	-	10.5 ± 0.7^b^	-	7.0 ± 0^b^
BLC29	18.5 ± 2.1^ab^	7.5 ± 0.7^b^	9.5 ± 0.7^a^	-	-	-	9.0 ± 1.4^ab^
BLC30	-	-	-	-	-	-	-
BLC31	-	-	-	-	-	-	-

^1^Diameter of the inhibition zone (mm); (-) No inhibition; Values with different superscript letters differ significantly (*p* < 0.05).

Notable inhibitory effects were also recorded against:

- *Enterobacter cloacae* (51.6% of strains), with inhibition zones of 7.0 ± 0 mm to 9.5 ± 0.7 mm;
- *Escherichia coli* (29.0%), with zones ranging from 7.5 ± 0.7 mm to 14.5 ± 2.1 mm;
- *Salmonella enteritidis* (25.8%), with inhibition zones between 7.0 ± 0 mm and 9.5 ± 0.7 mm;
- *Listeria monocytogenes* (25.8%), with diameters ranging from 8.5 ± 0.7 mm to 11.0 ± 5.7 mm;
- *Pseudomonas aeruginosa* (41.9%), with zones from 7.0 ± 0 mm to 10.5 ± 0.7 mm;
- *Bacillus cereus* (6.5%), with zones between 8.5 ± 0.7 mm and 9.0 ± 1.4 mm.

These findings support the antimicrobial potential of several LAB strains isolated from DCM, with implications for biopreservation and functional food applications.

### 3.11 Visual summary of technological and antimicrobial traits

**Figure 7:**
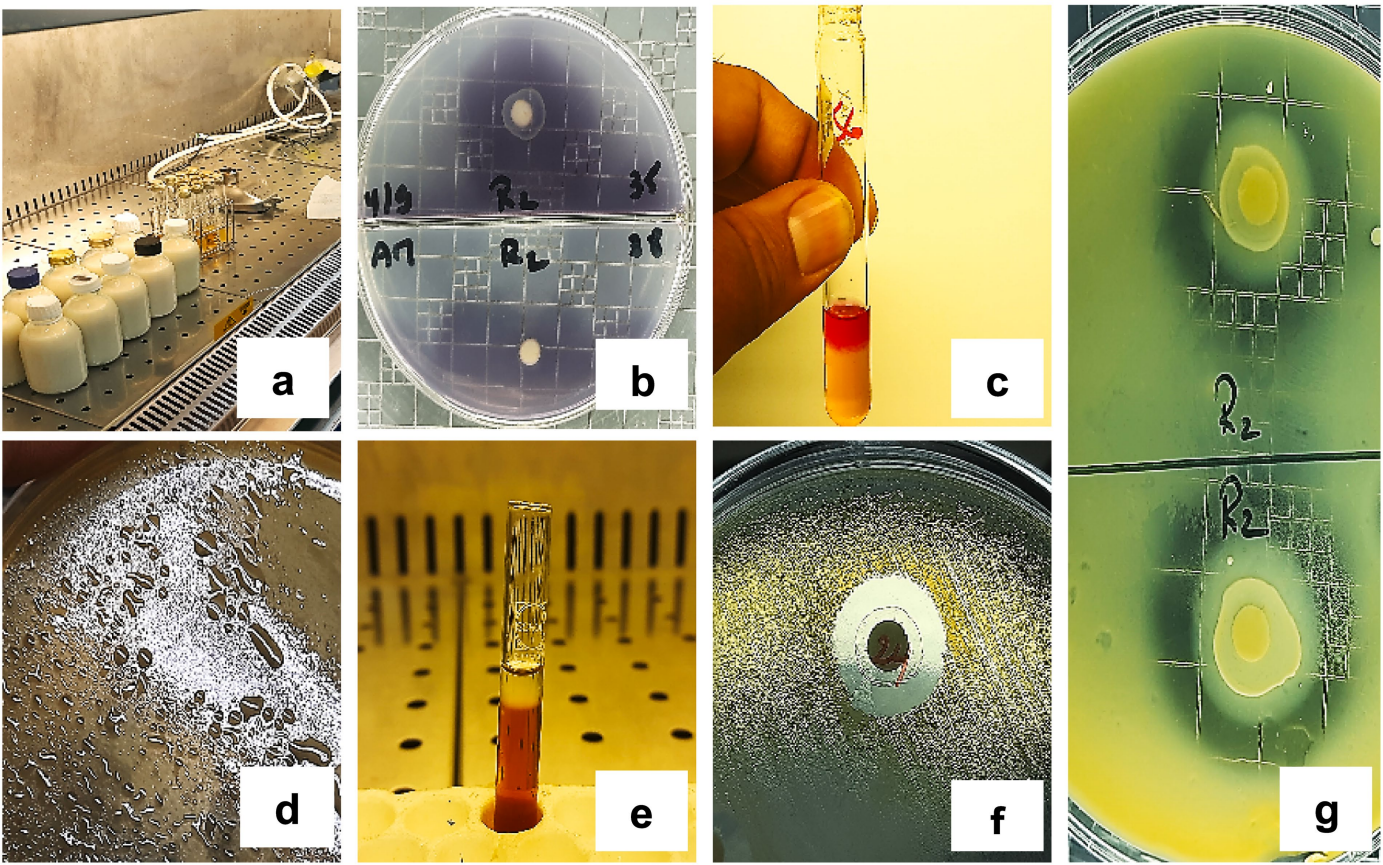
Representative results of the technological and antimicrobial activities of LAB strains. **(a)** Acidification activity, **(b)** Amylase production, **(c)** Acetoin production, **(d,e)** EPS production, **(f)** Antimicrobial activity, **(g)** Proteolytic activity.

The key technological and antimicrobial activities observed in the LAB isolates are visually summarized in Figure 7. Figure 7a shows acidification activity in milk culture tubes, while Figure 7b illustrates amylase activity evidenced by clear halo formation around inoculated discs on starch agar. Acetoin production is demonstrated in Figure 7c by the appearance of a pink ring in the Voges-Proskauer test. Figures 7d,e display exopolysaccharide (EPS) production, with visible slimy colonies and precipitate formation following ethanol addition. Figure 7f shows a representative inhibition halo indicating antimicrobial activity, and Figure 7g demonstrates proteolytic activity through casein hydrolysis on milk agar. Figure 7 provides qualitative confirmation of the diverse functional profiles detected across the LAB isolates.

### 3.12 Cluster analysis of technological and antimicrobial properties

**Figure 8:**
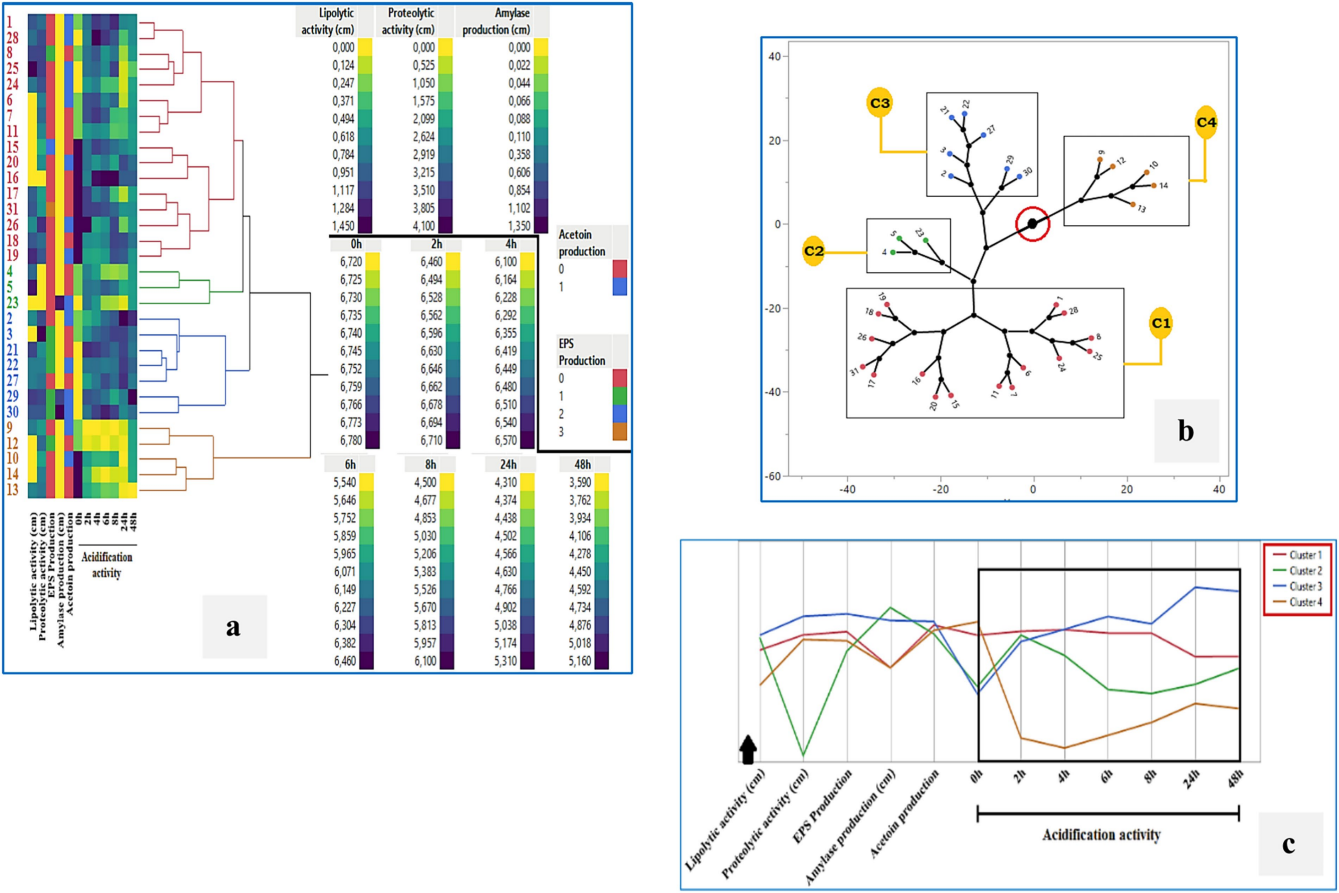
Cluster analysis of the technological activities of 31 LAB strains isolated from raw DCM samples: **(a)** Hierarchical clustering (Ward’s method), **(b)** Constellation plot, **(c)** Cluster profile overview. Cluster 1: BLC1, 6, 7, 8, 11, 15, 16, 17, 18, 19, 20, 24, 25, 26, 28, 31; Cluster 2: BLC4, 5, 23; Cluster 3: BLC2, 3, 21, 22, 27, 29, 30; Cluster 4: BLC9, 10, 12, 13, 14.

Hierarchical cluster analysis (HCA) using Ward’s method was performed to classify the 31 LAB isolates based on their technological and antimicrobial properties (Figures 8a–c). The heatmap (Figure 8a) illustrates the variability across strains in key parameters including lipolytic, proteolytic, and amylolytic activity, acidification over time, EPS and acetoin production. The HCA dendrogram revealed four distinct clusters with different functional profiles. The constellation tree (Figure 8b) confirmed these groupings, with Cluster 1 comprising the largest number of isolates that shared similar acidification and enzymatic traits. Cluster 3 included strains with minimal or no antimicrobial activity, such as *BLC4*, *BLC15*, *BLC17*, *BLC21*, *BLC23*, *BLC26*, *BLC30*, and *BLC31*, while Clusters 2 and 4 contained isolates with stronger inhibitory profiles. This grouping was further visualized in the line graph (Figure 8c), which showed how the average performance of each cluster differed across the measured variables. These results highlight the functional heterogeneity within the LAB population and suggest the presence of specific subgroups with enhanced biotechnological or biopreservative potential.

### 3.13 Cluster analysis of antibacterial activity

**Figure 9:**
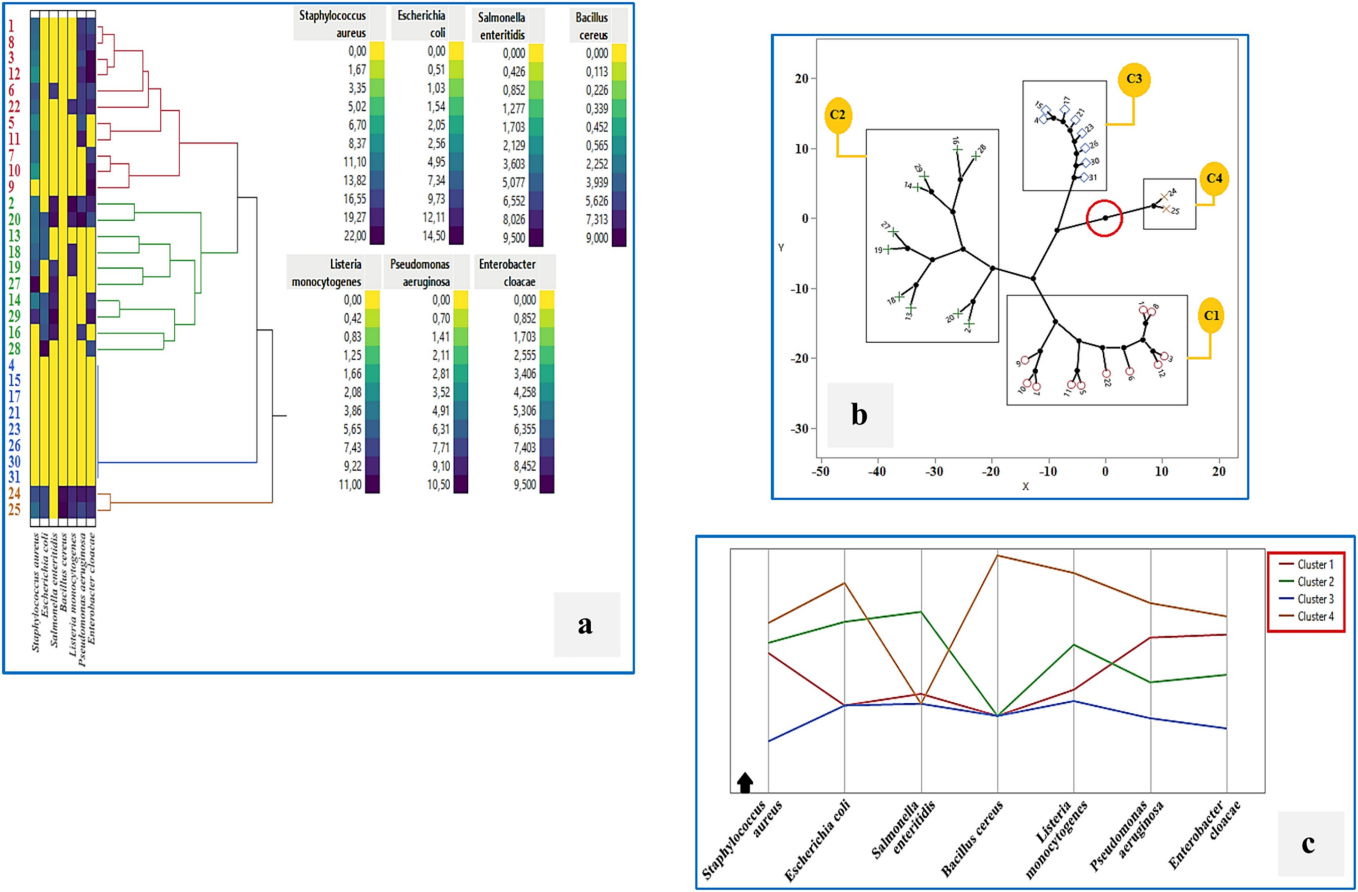
Cluster analysis of the antimicrobial activity of 31 LAB strains isolated from raw DCM samples: **(a)** Hierarchical clustering (Ward’s method), **(b)** Constellation plot, **(c)** Cluster profile overview. Cluster 1: BLC1, 3, 5, 6, 7, 8, 9, 10, 11, 12, 22; Cluster 2: BLC2, 13, 14, 16, 18, 19, 20, 27, 28, 29; Cluster 3: BLC4, 15, 17, 21, 23, 26, 30, 31; Cluster 4: BLC24, 25.

To further explore the functional diversity among LAB strains, hierarchical cluster analysis (HCA) was performed based on their antimicrobial activity profiles against seven foodborne pathogens (Figures 9a–c). Four major clusters were identified: Cluster 1 grouped strains with low to moderate antimicrobial activity, including *BLC1*, *BLC3*, *BLC5–12*, and *BLC22*. Cluster 2 contained strains with selective inhibition capacity (*BLC2*, *BLC13*, *BLC14*, *BLC16*, *BLC18*, *BLC19*, *BLC20*, *BLC27*, *BLC28*, *BLC29*). Cluster 3 included strains with minimal or no activity (*BLC4*, *BLC15*, *BLC17*, *BLC21*, *BLC23*, *BLC26*, *BLC30*, *BLC31*). Cluster 4, although small, included strains with strong and broad-spectrum antimicrobial activity (*BLC24* and *BLC25*). The constellation plot (Figure 9b) visualizes the distribution and relative distance of these clusters based on inhibition spectra, while the line graph (Figure 9c) highlights variability in pathogen-specific inhibition across clusters. Cluster 4 stood out with consistently high inhibition values against most pathogens, particularly *Staphylococcus aureus* and *Listeria monocytogenes*, suggesting potential application in food biopreservation. These findings confirm that specific LAB strains from Algerian DCM exhibit promising and differentiated antimicrobial potential.

## 4 Discussion

LAB are indispensable biotechnological tools in the global food industry. Their broad application in the fermentation of dairy, bakery, meat, and beverage products is supported by their GRAS (Generally Recognized as Safe) and QPS (Qualified Presumption of Safety) status, confirming their safety for human consumption (34). In this study, *Lactococcus lactis* dominated the LAB community (87.10%) in DCM, followed by *Enterococcus italicus* (6.45%), *Leuconostoc mesenteroides* (3.23%), and *Lactobacillus lactis* (3.23%). These findings align with previous investigations in Algeria, Morocco, Kuwait, and India (11, 35–37), confirming the prevalent LAB genera in camelid milk. Notably, this is the first report of *Enterococcus italicus* isolated from Algerian DCM, suggesting an original finding. Identification of the 31 LAB strains by MALDI-TOF mass spectrometry revealed a strong correlation with the results of phenotypic characterization. This technique has emerged as a rapid, accurate, and cost-effective method for bacterial identification, and its potential to replace classical phenotypic approaches in various microbiological applications is increasingly recognized (31, 38). In support of this, Dogan and Ozpinar (39) successfully identified 144 probiotic LAB strains from 130 food samples, including boza, cheese, kefir, and raw milk, in Turkey. Similarly, Gantzias et al. (40) reported a 95.5% identification rate of 88 non-starter LAB isolates from 18 artisanal Greek cheese samples, covering key species such as *Lactococcus lactis*, *Leuconostoc mesenteroides*, *Lactobacillus brevis*, *L. plantarum*, *L. rhamnosus*, *L. paracasei*, *Enterococcus faecium*, and *Pediococcus pentosaceus*. Among the tested strains, three *Lactococcus lactis* isolates exhibited strong acidifying capabilities *in vitro*, while four strains, including *Lactococcus lactis* and *Enterococcus italicus*, showed a slower acidification kinetic. The remaining isolates were classified as moderate acidifiers. Rapidly acidifying strains are particularly well-suited as primary starters in dairy fermentation, whereas slow acidifiers may be better employed as adjunct cultures depending on their broader technological profiles (41). Our findings contrast with those of Saidi et al. (42), who reported low acidifying potential in LAB isolated from Algerian DCM, but are consistent with the observations of Fguiri et al. (43), who described marked variability in this trait. Such differences may be attributed to strain-specific metabolic pathways involved in the catabolism of carbon and nitrogen sources (25).

Technological properties, particularly proteolytic and lipolytic enzymatic activities, play a fundamental role in shaping the organoleptic qualities of fermented foods, influencing aspects such as ripening, texture, and flavor development. In the present study, the lipolytic activity of the LAB strains was generally low, with inhibition halos measuring less than 1.5 cm (44). This modest lipolytic potential is desirable in cheese production, as it ensures a controlled release of free fatty acids, which are essential for flavor development without leading to rancidity (45). Similar low levels of lipolytic activity in LAB were reported by Davis et al. (46) in isolates from ewe’s milk cheeses, reinforcing the consistency of these findings. In addition, strains with reduced lipolytic activity can still effectively contribute to the sensory complexity of fermented dairy and meat products over extended ripening periods (44, 47). Regarding proteolytic activity, Vuillemard et al. (48) proposed that lysis zones between 1.5 and 2.1 cm serve as reliable indicators. Although most of our strains did not strictly fall within this range, substantial proteolytic activity was observed, notably in strain *BLC3*, which produced a halo measuring 4.10 ± 0.57 cm (25). Our findings are in line with previous work on LAB isolated from Algerian DCM, which highlighted strong proteolytic potential in strains such as *Lactococcus lactis*, *Enterococcus faecium*, *Lactobacillus plantarum*, and *Lactobacillus rhamnosus* (49). These results also corroborate other investigations across various LAB species (22, 50). The proteolytic system of LAB, including a suite of intracellular peptidases, plays a pivotal role in breaking down milk proteins into peptides and free amino acids that not only enhance taste but also serve as key precursors for aromatic compounds. Upon cell lysis, these enzymes are released into the matrix, further enriching the sensory profile of the final product (51).

Regarding EPS production, one strain, *Leuconostoc mesenteroides* (*BLC15*), exhibited moderate EPS synthesis, while *Lactococcus lactis* (*BLC31*) stood out for its high EPS yield, evidenced by large, slimy, and viscous colonies. These findings are in line with the results of Benhouna et al. (26), who demonstrated that LAB strains from traditional Algerian dairy products can hydrolyze sucrose and synthesize EPS. Similarly, Patel and Prajapati (52) identified *Streptococcus, Lactobacillus*, *Lactococcus*, *Leuconostoc*, and *Pediococcus* as the major EPS-producing genera among LAB. Notably, *Weissella* and *Leuconostoc* were reported to generate the highest dextran yields. Enhancing EPS productivity requires a deeper understanding of LAB biosynthetic metabolism and genetic regulation (53, 54).

Amylolytic LAB, capable of hydrolyzing starch into fermentable sugars and producing lactic acid, are essential in the fermentation of cereal-based products, where they influence both texture and flavor (55). In our study, only three strains demonstrated measurable amylolytic activity. This observation aligns with existing reports suggesting that LAB from camel milk exhibit limited starch-degrading capacity. Research in this area remains scarce, although Rao et al. (56) recently reported that only 5 out of 76 LAB strains isolated from sheep milk showed detectable amylolytic activity, as indicated by halo formation around the colonies.

A substantial proportion, nearly half, of the tested LAB isolates demonstrated acetoin-producing fermentative pathways, highlighting their potential for technological applications in aroma development. This finding contrasts with an earlier study, which reported that only 2 out of 8 LAB strains isolated from DCM in southwestern Algeria were capable of producing diacetyl and acetoin (35). By comparison, Domingos-Lopes et al. (22) found high acetoin-producing activity among LAB strains isolated from traditional raw cow’s milk cheeses from Pico Island in the Azores, with notable frequencies in *Leuconostoc* (60%), *Lactococcus* (33%), *Lactobacillus* (82%), and *Enterococcus* (92%). Acetoin, a secondary metabolite resulting from the oxidative decarboxylation of *α*-acetolactate, contributes significantly to the aroma profile of fermented foods. It frequently coexists with diacetyl, a key compound responsible for buttery notes in beer, wine, dairy products, and bread. Among LAB, *Enterococcus* and *Lactobacillus* are particularly important contributors to acetoin production (22, 57).

Analysis of the inhibition spectra revealed notable antimicrobial activity in the majority of LAB strains tested (74.19%). *Staphylococcus aureus* was particularly susceptible, with strain *BLC27* exhibiting the strongest inhibitory effect, characterized by a zone of inhibition measuring 22.0 ± 2.8 mm. In contrast, eight out of the 31 isolates showed no detectable antimicrobial activity, while *Bacillus cereus* exhibited partial resistance to the bioactive compounds produced. The application of non-pathogenic microorganisms such as LAB for food biopreservation has gained increasing attention, given their ability to suppress undesirable microbes and extend product shelf life. Their production of antimicrobial and antioxidant metabolites not only enhances microbial safety but also contributes to the nutritional and sensory quality of foods, with promising applications in the cosmetic and pharmaceutical sectors as well (58, 59). Eddine et al. (60) demonstrated the broad-spectrum antimicrobial potential of LAB isolated from DCM in the arid regions of southern Algeria, with some strains showing strong inhibition against *Pseudomonas aeruginosa* (22 ± 1.00 mm) and others active against *E. coli* and *S. aureus*. A recent study on LAB isolated from camel milk in Faisalabad, Pakistan, revealed variable inhibitory activities among the strains. The *Lactobacillus casei*-04 strain demonstrated an inhibition zone of 15.33 ± 0.58 mm against *Escherichia coli* AZ1. On the other hand, the *Lactobacillus casei*-05 strain exhibited a maximum inhibition zone of 16.33 ± 1.15 mm against *Staphylococcus aureus* Saba-1 (61). These findings are consistent with other studies reporting similar antimicrobial capacities in LAB isolates (59, 62, 63), underscoring their value as natural biopreservatives. Finally, hierarchical cluster analysis (HCA) was used to explore functional relationships among the 31 LAB isolates, resulting in two distinct classification schemes: one based on technological parameters (Figure 8) and the other on antimicrobial activity (Figure 9). In the technological cluster analysis, four main groups emerged, with Cluster 1 encompassing the largest number of strains that shared moderate acidification, proteolytic activity, and low amylolytic or EPS production. Cluster 3, in contrast, contained strains with minimal or absent technological traits, reflecting limited suitability for fermentation applications. When clustering was based on antibacterial activity, a different pattern emerged. Cluster 4, although small, was notable for comprising strains (*BLC24* and *BLC25*) with broad-spectrum and high-level inhibitory activity against foodborne pathogens, including *Staphylococcus aureus*, *Listeria monocytogenes*, and *Escherichia coli*. Conversely, Cluster 3 of the antimicrobial profile included strains with no measurable activity against the tested pathogens, limiting their relevance in food safety contexts. Together, these complementary clustering approaches underscore the functional heterogeneity of LAB populations from Algerian DCM and help identify promising candidates for targeted industrial use, whether for starter culture development, functional food enhancement, or natural biopreservation strategies.

## 5 Conclusion

This study revealed substantial inter-strain variability in the technological and functional attributes of LAB isolated from DCM. Several strains demonstrated promising acidifying and proteolytic capacities, along with the ability to produce exopolysaccharides (EPS) and acetoin, traits that are desirable for fermented food applications. In contrast, lipolytic and amylolytic activities were generally low across the collection. Importantly, a majority of the isolates exhibited significant antimicrobial activity, underscoring their potential as natural biopreservatives in food systems. These findings position DCM from the semi-arid regions of Algeria as an untapped ecological niche rich in functionally diverse LAB strains with both technological and bioconservative potential. This work provides an original scientific contribution to the characterization of LAB from a unique ecosystem and lays the foundation for future applied research. Ongoing investigations aim to validate the efficacy of selected strains in real food matrices, particularly in response to the growing demand for clean-label products free from synthetic additives.

## Data Availability

The original data presented in the study are included in the article, further inquiries can be directed to the corresponding author.
